# Emerging Trends in Left Ventricular Thrombus: A Comprehensive Review of Non-Ischemic and Ischemic Cardiopathies, Including Eosinophilic Myocarditis, Chagas Cardiomyopathy, Amyloidosis, and Innovative Anticoagulant Approaches

**DOI:** 10.3390/diagnostics14090948

**Published:** 2024-04-30

**Authors:** Benjamin Colle, Fabian Demeure, Julien Higny, Martin Benoit, Jean-Philippe Henry, Isabelle Michaux, Benoit Robaye, Olivier Xhaët, Laurence Gabriel, Antoine Guedes, Dominique Blommaert, Nathalie Dulieu, Yannick Berners, Fabian Wery, Steven Droogmans, Bernard Cosyns, Maria-Luiza Luchian

**Affiliations:** 1 Department of Cardiology, Université Catholique de Louvain (UCL), CHU UCL Namur Site Godinne, Av. Dr. G. Thérasse, 1, 5530 Yvoir, Belgium; 2 Department of Intensive Care, Université Catholique de Louvain (UCL), CHU UCL Namur Site Godinne, Av. Dr. G. Thérasse, 1, 5530 Yvoir, Belgium; 3 Department of Cardiology, Centrum voor Hart-en Vaatziekten, Universitair Ziekenhuis Brussel, Vrije Universiteit Brussel (VUB), Laarbeeklaan 101, 1090 Brussels, Belgium

**Keywords:** left ventricle thrombus, echocardiography, amyloidosis, ischemic heart disease, anticoagulation, hypertrophic cardiomyopathy, apical aneurysm, left ventricle dysfunction

## Abstract

This comprehensive review explores the intricate aspects of left ventricular thrombus (LVT), a potential complication in both ischemic and non-ischemic cardiomyopathies. It provides a thorough understanding of left ventricular thrombus, revealing its uncommon incidence in the general population (7 cases per 10,000 patients), predominantly linked to ischemic heart diseases (ICMs) at an 80% prevalence rate. Diagnostic tools, notably transthoracic echocardiography (TTE) and cardiac magnetic resonance imaging (CMR), demonstrate varying sensitivity but remain indispensable in specific clinical contexts related to LVT as non-invasive diagnostic modalities. A detailed comparison between ICM patients and those with non-ischemic cardiomyopathy (NICM) who have left ventricular thrombus reveals subtle distinctions with significant clinical implications. This analysis underscores the importance of these imaging techniques in distinguishing between the two conditions. Additionally, we explored the occurrence of LVT in specific non-ischemic cardiomyopathies, including Takotsubo syndrome, hypertrophic cardiomyopathy, eosinophilic myocarditis, Chagas disease, cardiac amyloidosis, and several other conditions. The article further delves into anticoagulation strategies, thoroughly examining their impact on LVT regression and patient outcomes. Pharmacological interventions, with a focus on direct oral anticoagulants, emerge as promising alternatives; however, there is insufficient information on their efficiency and safety, especially in NICM population. In conclusion, this review highlights the complex nature of LVT, incorporating a range of etiopathogenic factors, diagnostic complexities, and evolving therapeutic approaches. It emphasizes the pressing need for ongoing research in this field.

## 1. Introduction

Left ventricular thrombus (LVT) represents a potential complication observed in both ischemic (ICM) and non-ischemic cardiomyopathies (NICM), posing an additional risk of thromboembolic events, including strokes.

The aim of the present review is to address the epidemiology and pathophysiology of LVT formation, as well as the advantages and limitations of the current available cardiovascular imaging diagnostic modalities for LVT detection and its management strategies. Furthermore, we analyze the current data on epidemiological differences between ICM and NICM patients with LVT. The epidemiology of different non-ischemic cardiomyopathies leading to ventricular thrombus—Takotsubosyndrome, hypertrophic cardiomyopathy (HCM) associated with apical left ventricular aneurysm, eosinophilic myocarditis, Chagas disease cardiomyopathy, and cardiac amyloidosis (CA)—are further explored. Additionally, this exploration extends to other medical conditions such as chemotherapy-induced cardiopathy and peripartum cardiopathy.

Finally, LVT therapeutic management with a focus on the role of direct oral anticoagulants is examined.

An extensive review of the literature derived from research involving human subjects, published in English and indexed in MEDLINE (through PubMed), was conducted. Key search words included but not limited to were the following: left ventricle thrombosis and myocarditis, thrombus and myocardial injury, acute coronary syndrome, myocardial infarction and left ventricle thrombus, Takotsubo cardiomyopathy, Chagas disease cardiomyopathy, left ventricle thrombus and anticoagulation, left ventricle aneurysm, amyloidosis, eosinophilic myocarditis, apical left ventricle hypertrophy, left ventricle thrombus and cardiac magnetic resonance.

## 2. Epidemiology of Left Ventricular Thrombus and Pathophysiology

**Figure 1:**
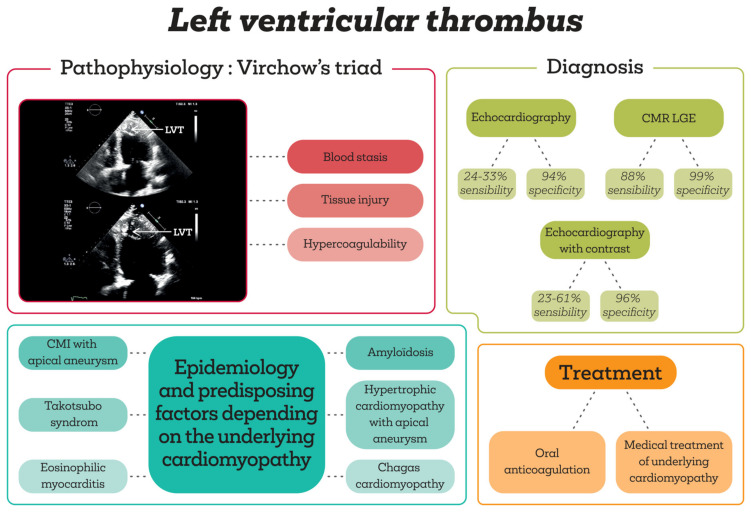
The background mechanism of left ventricle thrombus (LVT) formation in both ischemic and nonischemic cardiomyopathy is represented by the presence of left ventricle dysfunction in the context of multifactorial myocardial injury. Cardiac magnetic resonance (CMR) with late gadolinium enhancement (LGE) evaluation constitutes the gold standard for LVT detection; however, the accessibility is limited. Therefore, echocardiography with or without contrast is routinely performed nowadays in the case of LVT suspicion. Currently, the medical management includes anticoagulation in addition to the specific treatment of the underlying cardiomyopathy.

The overall LVT prevalence in the general population is low. In a retrospective review of over 80,000 medical records, the LVT incidence was 7 per 10,000 patients [1]. Most cases were associated with ischemic heart disease (80%), especially after anterior myocardial infarction, while the remainder were attributed to dilated cardiomyopathy (DCM) and stress-induced cardiomyopathy (8.15% and 4.8%, respectively) [1,2]. Studies from the pre-percutaneous coronary intervention and pre-thrombolytic era report an LVT prevalence ranging from 28% to 46% in anterior myocardial infarction [3,4,5,6]. In the era of primary percutaneous coronary intervention (PCI), depending on the diagnostic modalities LVT prevalence varies from 5% to 15% when assessed using transthoracic echocardiography (TTE) [6,7,8] to 12% to 27% using cardiac magnetic resonance (CMR) imaging evaluations [9,10,11]. Risk factors associated with LVT occurrence are traditionally encompassed in Virchow’s triad [1,12]. Incorporating myocardial tissue damage, this concept applies to various diseases, including myocardial infarction, HCM, Chagas disease, and certain immune disorders like hypereosinophilic syndrome and Behcet’s disease (Figure 1—Left Ventricle Thrombus from Pathophysiology to Diagnosis and Medical Management).

The presence of abnormal blood flow is particularly observed in situations involving severe left ventricular dysfunction or when the left ventricular apex is affected by an anterior–apical scar after a myocardial infarction [6]. This is also seen in cases of Takotsubo syndrome with an apical form [13,14] or HCM with an apical aneurysm. Notably, severe left ventricular dysfunction is a critical predictive indicator for LVT occurrence [6,15] accompanied by a hypercoagulability state, as observed in chronic heart failure [16,17].

### 2.1. LVT Formation: Prevalence Pre- and Post-PCI

The intensity of myocardial damage is linked to the susceptibility of intraventricular thrombus formation, especially after an acute ischemic event. In the context of an ICM, the risk of LVT occurrence depends on the symptomology onset and the time to treatment [18]. The timing of cardiac imaging evaluation after a myocardial infarction plays a crucial role in identifying those patients at risk of LVT formation, which may alter their short- and long-term prognoses. Despite the advances in cardiac imaging modalities and the current treatment options, most studies underline the same message: the true incidence of LVT is unknown and probably overlooked. In ICM, the factors affecting the formation of intraventricular thrombus are associated with the extent of the infarction area. This is particularly evident in cases of anterior myocardial infarction, where significant regional wall abnormalities are observed [1]. Overall intraventricular thrombosis prevalence surpasses 10% in the cases of anterior infarction, as previously stated [3,4,5,19,20,21], being less frequently described in other localizations, e.g., inferior myocardial infarction. Nevertheless, most of the data come from studies performed during the pre-PCI era. Contrastingly, increased access to PCI, reduced timing from the symptomatology onset, and invasive treatment modifies the risk of LVT formation in the context of an acute myocardial infarction; however, it does not cancel it. A retrospective study performed during the PCI era comprising more than 150,000 patients with anterior myocardial infarction with an elevation of the ST segment (STEMI) showed a prevalence of intraventricular thrombosis of 0.4%; however, LVT presence was associated with a 4-fold augmentation of thromboembolic events [22]. In terms of population characteristics, patients with anterior STEMI and LVT had a higher incidence of atrial fibrillation (16.9% vs. 12.4%; *p* < 0.001), aortic atherosclerosis (3.7% vs. 1.6%; *p* < 0.001), use of long-term anticoagulation (4.6% vs. 1.7%; *p* < 0.001), use of a Swan–Ganz catheter (9.4% vs. 5.7%; *p* < 0.001), intra-aortic balloon pump use (18.0% vs. 11.6%; *p* < 0.001), cardiogenic shock (17.9% vs. 10.4%; *p* < 0.001), and acute respiratory failure (23% vs. 13.7%; *p* < 0.001) compared to patients without LVT [22]. Conversely, there was no difference in terms of medical strategy (PCI versus coronary artery bypass grafting) and the occurrence of intraventricular thrombosis [22]. However, the average duration and costs of hospitalization and subsequent follow-ups were considerably higher in this population [22]. Additional research has documented a heightened LVT frequency persisting into the period of widespread PCI use. A prospective study with a cohort of 100 consecutive patients with a left ventricle ejection fraction under 45% and anterior myocardial infarction, showed a 26% occurrence of LVT after PCI, diagnosed at a median of 12 days [23]. The investigators performed three TTE evaluations for intraventricular thrombus detection. LVT was detected in the first 6 weeks after myocardial infarction and completely resorbed at day 270 [23], emphasizing the importance of long-term follow-up of those patients.

### 2.2. LVT Formation: Mechanisms beyond Myocardial Infarction

The development of intraventricular thrombosis is associated with the components of Virchow’s triad, which includes endothelial damage due to myocardial infarction, blood stasis resulting from left ventricular dysfunction, and increased coagulability initiated via inflammation [20]. Patients with LVT have a prolonged and reduced wash-in of LV in comparison to healthy individuals in whom the blood remains shortly in the LV cavity in diastole due to high intraventricular pressure gradients [24]. Left ventricle regional thinning and dilation with damaged endothelium are associated with increased wall stress [18]. These structural abnormalities lead to blood stasis, primarily driven by left ventricular dysfunction, marked by reduced ejection fraction and abnormal vortex formation due to impaired contractile function [18]. Endothelial injury induces inflammation and a prothrombotic state by exposing subendothelial tissue, promoting hypercoagulability [25]. Baseline C-reactive protein, fibrinogen, and the neutrophil–lymphocyte ratio independently predict early LVT formation after myocardial infarction [18].

Each of these factors represents possible targets for therapeutic intervention. The core knowledge concerning tissue injury, stasis, and fibrin cross-linking in thrombus formation justifies the preference for anticoagulants over antiplatelet drugs in patients with left ventricular thrombus.

### 2.3. Diagnostic Modalities in Left Ventricular Thrombus Detection

The 2016 recommendations from the American Society of Echocardiography regarding TTE use in evaluating cardiac sources of embolism describe the latter as the technique of choice and the most widely used in clinical settings. It is employed in assessing the regional and global function of the left and right ventricles, valvular evaluation, and LVT assessment. LVT is identified as a discrete echocardiographic mass seen in the left ventricle with well-defined margins distinct from the endocardium, visible throughout the systole and diastole in an area with significant abnormalities of the left ventricular wall, either regional or global [26] (Figure 1). To confirm the diagnosis, it should be visualized in at least two orthogonal views (apical and short-axis). The role of transesophageal echocardiography in LVT diagnosis is limited due to an often shortened and/or poorly visualized left ventricle.

The accuracy of LVT diagnosis using TTE exhibits significant variability. In a prospective study involving 100 patients, the focus was on assessing the occurrence and progression of LVT in a high-risk population following anterior myocardial infarction and associated left ventricular systolic dysfunction [23]. Among these patients, 26 were identified with left ventricular thrombi, typically appearing around 12 days after anterior myocardial infarction [23]. To confirm these findings, all participants in the study underwent late gadolinium-enhanced cardiac magnetic resonance (CMR LGE) [23]. The sensitivity and specificity of transthoracic echocardiography were 94.7% and 98.5%, respectively, compared to CMR LGE [23].

A systematic review involving over 800 patients, evaluating TTE accuracy in comparison to CMR examinations for LVT detection, revealed the superiority of CMR across different acquisition protocols. CMR, specifically using LGE protocols, demonstrated the highest accuracy (sensitivity: 88%; specificity: 99%), followed by cine-CMR (sensitivity: 58%; specificity: 99%; accuracy: 95%; positive predictive value: 93%–95%; negative predictive value: 95–96%) [27]. Contrast-enhanced TTE exhibited a sensitivity range of 23% to 61%, specificity between 96% and 99%, accuracy of 92%, a positive predictive value of 93%, and a negative predictive value of 91% [27]. Finally, contrast-free TTE showed a lower sensitivity, ranging between 24% and 33%, specificity between 94% and 95%, accuracy of 82%, a positive predictive value of 57%, and a negative predictive value of 85% [27]. The authors suggested that the accuracy of TTE could be improved with a clear clinical indication and the systematic use of left ventricular contrast agents [27]. In another meta-analysis assessing the true LVT incidence via CMR in acute myocardial infarction treated with PCI, encompassing ten studies and 2072 patients, TTE demonstrated a sensitivity of 29% and a specificity of 98% compared to CMR. The meta-analysis reported an overall LVT incidence of 6.3%, with 96% occurring in the context of anterior ST-segment elevation myocardial infarction [28]. Despite diagnostic superiority and being considered the gold standard, the limited availability and cost of CMR restrict its routine use. Some authors have suggested the use of an apical motion score to stratify patients requiring CMR in confirming LVT following a myocardial infarction. In a study by Weinsaft et al., the use of the apical motion score allowed all patients with LVT suspicion to be systematically referred to CMR evaluation for diagnostic confirmation, achieving a sensitivity of 100% while halving the use of other diagnostic tests [29]. Another aspect to be considered when analyzing the presence of LVT is the moment of evaluation, which depends on the underlying cardiopathy. The LVT occurrence is maximal within 9 to 12 days after myocardial infarction, while it peaks within 48 h in the context of Takotsubosyndrome with apical involvement [13,23,30].

Importantly, LVT may be misdiagnosed in the presence of important trabeculations, as previously described in noncompaction cardiomyopathy. The current guidelines do not consider the presence of hypertrabeculations asa distinctive cardiomyopathy [31]. However, it is important to mention, as they can be associated with LVT [31]; therefore, non-invasive cardiac imaging evaluation remains the cornerstone for proper management. Earlier recommendations papers reinforce the importance of CMR evaluation in patients’ hypertrabeculations. CMR provides superior resolution and tissue characterization properties compared to TTE examinations, and therefore, it should be the imaging modality of choice in patients with hypertrabeculations to accurately identify apical thrombi and endomyocardial fibrosis, aiding in effective thromboembolic risk assessment, if LVT is detected [32]. The treatment strategy continues to favor oral anticoagulation, with both strategies, direct oral or antivitamin K anticoagulation, being proposed [32]. In the absence of predisposing factors for an embolic event (e.g., atrial fibrillation, LVT presence) preventive anticoagulation therapy remains debatable. Patients with apical hypertrabeculations and left ventricular dysfunction in the presence of sinus rhythm are considered at a higher risk of thromboembolic events. This risk is particularly pronounced in those with severe systolic dysfunction and the presence of LGE on CMR examination, as they have been shown to experience more adverse cardiovascular events, including stroke [32,33]. Therefore, long-term anticoagulation, preferably with direct oral anticoagulants, is recommended for patients previously considered with noncompaction cardiomyopathy in the presence of significant LV dysfunction [32,34]. For individuals diagnosed with ‘noncompaction cardiomyopathy’ in the absence of atrial fibrillation, left ventricle systolic dysfunction, or intracardiac thrombi, recent findings indicate that the presence of an important ratio of apical hypertrabeculations may pose a risk of thromboembolic events, e.g., cryptogenic stroke, particularly in young patients without cardiovascular risk factors [32,33]. While guidelines do not currently recommend preventive therapy for thromboembolic events in these cases, anticoagulant therapy could be proposed for patients with a CHADS2 score of ≥2 [32,34].

## 3. Comparison between LVT in ICM and NICM

The majority of data on LVT pertain to ICM patients, with limited studies on the epidemiology, predictive factors, and secondary risks associated with LVT presence in NICM. A recent study by Hooks M. et al. compared a NCIM LVT population of 48 patients with 124 LVT ICM patients and 144 matched patients without left ventricular thrombus in NICM [15]. Compared to patients with thrombus in ICM, those with thrombus in NICM exhibited lower ejection fractions, larger left ventricles, fewer left ventricular aneurysms, lower LGE, and a smaller LGE extent. However, there were more left ventricular thrombi per patient, and these patients were ten times more likely to have a thrombus in intracardiac cavities other than the left ventricle (4% vs. 39.6% in ICM and NICM, respectively) [15]. The one-year risk of embolic events was not different between patients with ICM or NICM, with 6.8% of embolic events in ICM and 8.7% in NICM, significantly higher than in NICM patients without LVT, for whom the annual risk of embolic events was 1.5%. Patients with LVT in non-ischemic cardiomyopathy exhibit lower ejection fractions, larger left ventricles, a marked LGE prevalence, and a more extensive LGE extent compared to those without left ventricular thrombus in NICM [15]. In addition to assisting in identifying the precise cause of cardiomyopathy, CMR facilitates risk stratification for LVT’s onset based on LGE and its extent. In this study, the presence of LGE was associated with a sixfold higher probability of left ventricular thrombi.

### 3.1. Takotsubo Syndrome

Takotsubo syndrome (TTS) is characterized by acute left ventricular dysfunction, a factor that may contribute to LVT formation. In a recent study by Katharina J. Ding et al. [13], TTS patients enrolled in the International akotsubo Registry across 28 centers in Australia, Europe, and the United States were dichotomized based on the occurrence/absence of intraventricular thrombus or systemic embolism. Out of 1676 TTS patients, 56 (3.3%) developed intraventricular thrombus and/or systemic embolism [13]. The occurrence of left ventricular thrombus was early, with a median time interval of 2 days (range: 0–38 days) [13]. The clinical profile of patients with intraventricular thrombus and/or embolism was also different compared to patients without thrombus. Patients in the thrombus and/or embolism group had a more significant impairment of left ventricular ejection fraction, a higher prevalence of the apical type, higher levels of troponins and inflammatory markers (CRP and leukocytosis), and a higher prevalence of cardiovascular diseases [13]. These patients also experienced more complications, with a higher rate of cardiogenic shock, catecholamine use, and invasive or non-invasive ventilation [13]. A thrombus risk score (InterTAK Thrombus Risk score) was developed, consisting of four variables: the presence of an apical type (1.5 points), a history of vascular disease (1.5 points), a left ventricular ejection fraction below 30% at admission (1.5 points), and an initial white blood cell count > 10 × 10^3^ cells/μL (1 point) [13]. Patients with scores higher than 3 are at high risk of thrombus formation and/or embolism, with an incidence between 10.6% and 15.6% [13].

Comparable prevalence data were identified in another registry study by Santoro F. et al. [14]. Within the GEIST registry (German Italian Spanish Takotsubo registry) comprising 541 patients, 12 individuals with Takotsubo syndrome (TTS) exhibited LVT occurrence, resulting in a prevalence rate of 2.2% [14]. Notably, all 12 LVT patients were exclusively women with apical involvement [14]. Nevertheless, the true prevalence of LVT is yet undetermined.

The meta-analysis by Baldetti L et al. reported an LVT rate across various studies ranging from 1.3% to 7.7% with a combined estimated rate of 1.8% [35]. Within this meta-analysis, 83 patients out of a total of 3823 presented LVT [35].

### 3.2. Hypertrophic Cardiomyopathy with Apical Aneurysm

Several retrospective studies suggest an association between LVT and HCM with apical aneurysm in which its presence provides a structural nidus with abnormal blood flow favoring thrombus formation [31].

In a study led by Rowin et al. comprising 1940 HCM patients, including 93 individuals with left ventricular apical aneurysms (4.8%), 13 HCM patients (14%) with left ventricular apical aneurysm exhibited LVT without associated thromboembolic events [36]. Thrombi were identified via CMR or cardiac computed tomography (n = 6), contrast echocardiography (n = 4), or both echocardiography and CMR (n = 3) [36]. The size of the aneurysm, defined as the maximal transverse dimension measured on CMR or TTE and categorized as small (<2 cm), medium (2 to 4 cm), or large (>4 cm), seems to be an important factor in thrombus occurrence or embolic events, with the majority occurring in medium to large aneurysms (14 out of 18 patients, 78%) [36]. Patients presenting LVT or embolic events were subsequently treated with anticoagulants (mainly warfarin), and none experienced thromboembolic events over an average of 4.0 ± 2.8 years [36]. The investigators suggested prophylactic anticoagulation in HCM patients with apical aneurysm at risk of LVT development. To support this, a recent meta-analysis of six studies, including the study by Rowin et al., showed that the likelihood of an embolic event is 6.30 times higher compared to HCM patients without a left ventricular apical aneurysm [37].

Another study by Lee D. et al. addressed a similar hypothesis by evaluating 160 HCM patients with apical aneurysm [38]. Over an average follow-up duration of 6.2 years ± 4.8 years, 23 patients (15%) were diagnosed with LVT through CMR or TTE [38]. Those with an aneurysm larger than 2 cm faced an almost fourfold higher risk of LVT developing. Notably, five of these patients experienced both LVT and a stroke [38]. Interestingly, this study did not find a significant correlation between aneurysm size and the risk of stroke.

However, a noteworthy number of patients with moderate to large aneurysms were on anticoagulant therapy, potentially averting stroke incidents based on the identification of apical thrombus. Patients with an aneurysm larger than 2 cm had a 2.2 times higher relative risk of developing a stroke or LVT compared to patients with an aneurysm smaller than 2 cm [38]. These data also suggest the detrimental role of aneurysm size in prognosis and raise the question of initiating anticoagulation for an aneurysm size greater than 2 cm [38].

Across all of these studies, HCM patients and left ventricular apical aneurysm exhibited an increased risk of major cardiovascular events, such as sudden cardiac death or end-stage heart failure, highlighting that this subgroup presents a heightened risk of adverse events.

### 3.3. Eosinophilic Myocarditis

Acute myocarditis is defined according to the latest European consensus published by Caforio et al. in 2014 as an inflammatory disease of the myocardium diagnosed based on histological evidence of inflammatory infiltrates in the myocardium associated with myocyte degeneration and non-ischemic necrosis, immunological, and immunohistochemical criteria [39]. This definition includes the need for endomyocardial biopsies to confirm the diagnosis and underlying etiology (lymphocytic myocarditis, eosinophilic myocarditis, giant cell myocarditis, and granulomatous myocarditis). Eosinophilic myocarditis (EM) is a relatively rare form associated with parasitic infections, hypersensitivity reactions to various agents, eosinophilic inflammatory disorders, and, rarely, a neoplastic process [40]. It is also associated with LVT occurrence [40].

A significant number of publications detailing the link between LVT and EM are in the form of case reports or case series. In a systematic review with the objective of outlining the clinical presentation, treatment, and outcomes of EM, 179 patients with histologically confirmed EM were examined [41]. Twenty-two patients out of one hundred and sixty-one with complete cardiac imaging evaluation were identified to have LVT, resulting in a prevalence of 13.6% [41]. The analyses were also subdivided based on the underlying condition causing EM, EM related to hypersensitivity, EM related to eosinophilic granulomatosis with polyangiitis (EGPA), EM related to complex hypereosinophilic syndrome, and EM related to idiopathic causes. The risk of developing LVT is mainly found in patients with complex hypereosinophilic syndrome and EGPA, with a prevalence of thrombus occurrence of 28.6% and 19%, respectively, in this population [41].

### 3.4. Chagas Cardiomyopathy

Chagas disease is an acute or chronic infection caused by the flagellated protozoan *Trypanosoma cruzi* (*T. cruzi*) [42]. *T. cruzi* infection is predominant in regions of South America, where it is estimated that up to 8% of the population is seropositive, but only 10 to 30% will develop symptomatic disease [42,43,44]. Ten to twenty years after infection, irreversible cardiac lesions may appear. The main manifestations of chronic Chagas cardiomyopathy are heart failure, arrhythmia, and sudden cardiac death [42,45]. Chagas cardiomyopathy has also been independently associated with the risk of developing a stroke, with a significant cardioembolic risk secondary to the atrial fibrillation or LVT occurrence [42,45,46]. A recent study by Moreira HT et al., involving 330 patients with Chagas cardiomyopathy, found a prevalence of ischemic cerebrovascular events (ICE) of 20%, with 67 patients experiencing an ICE [46]. Most patients were classified in New York Heart Association classes I or II (75%) with an average left ventricular ejection fraction of 39 ± 14% [46]. Left ventricular mural thrombi were found in 48 patients (15%), and 128 patients (39%) had apical aneurysms. Multivariate analysis, including potential predictors of ICE, identified apical aneurysm and LVT as important determinants of ICE after adjustment for anticoagulant therapy [46], similar to HCM with apical aneurysm.

### 3.5. Cardiac Amyloidosis

CA may lead to a significant impairment of cardiac function, and one of its potentially fatal complications is the formation of intracardiac blood clots. While a few cases of intraventricular thrombus have been described, it is important to note that, in most cases of amyloidosis, intracardiac clots are primarily found in the atrial appendages. A retrospective study by Feng et al., involving 159 CA patients with transesophageal echocardiography evaluation, revealed a prevalence of 27% for intracardiac thrombosis, with almost all found in the atrial appendages (88%) [47]. There are currently no available data on the actual LVT prevalence in CA patients. A study conducted by Martinez-Naharro A. et al. on 324 CA-diagnosed patients revealed a notable prevalence of intracardiac thrombi in patients with both cardiac amyloidosis and atrial fibrillation even with proper anticoagulation (13.1% of study patients), challenging current cardioversion guidelines [48]. The authors proposed the necessity of tailored cardiac imaging in CA patients prior to cardioversion, regardless of their anticoagulation status. This is reinforced by multiple case reports indicating a heightened risk of intracardiac thrombi, even in the absence of documented atrial fibrillation, within this unique patient cohort. Hence, the question of whether prophylactic anticoagulation is universally warranted in all CA patients remains open, necessitating further research.

### 3.6. Left Ventricle Thrombosis in the Cardio-Oncology Era

**Figure 2:**
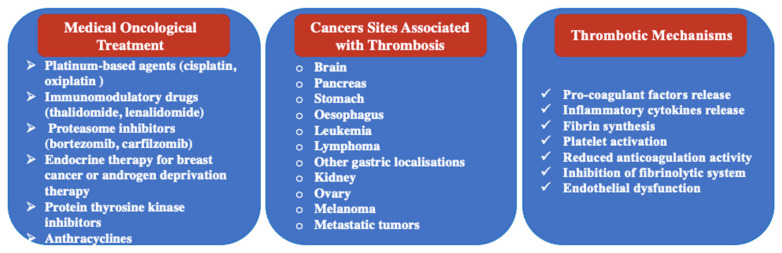
Oncological therapies and cancer localizations associated with high incidence of thrombotic events. Intrinsic mechanisms triggering thrombosis vary from activation of pro-coagulant factors and process to inhibition of intrinsic anticoagulation action.

The risk of thrombosis is influenced by the extent of coagulation abnormalities and endothelial damage caused by both cancer itself and the treatment used, e.g., chemotherapy with platinum compounds (e.g., cisplatin and oxiplatin), immunomodulatory drugs (e.g., thalidomide and lenalidomide), proteasome inhibitors (e.g., bortezomib and carfilzomib), endocrine therapy for breast cancer, or androgen deprivation therapy (Figure 2) [49,50]. Despite its infrequency, multiple case reports have underscored the significance of screening for intracardiac thrombi, given the high-risk population and potential life-threatening consequences [51,52].

Determining the LVT incidence in chemotherapy-associated cardiac dysfunction remains challenging, primarily due to limited sample sizes in studies, the predominance of retrospective designs, and variations in research methodologies. For example, in a study of 121 patients with chemotherapy-related severe LV dysfunction (LV ejection fraction ≤ 30%), 9 (7.4%) patients were diagnosed with intracardiac thrombi [53].

The thrombotic mechanisms in cancer patients differ from those without a history of cancer, with the tissue factor playing a pivotal role in cancer-associated thrombosis by activating the extrinsic coagulation pathway, leading to fibrin synthesis and platelet activation [54]. Moreover, cancer cells may produce cancer pro-coagulant factors and inflammatory cytokines, promoting endothelial dysfunction and further contributing to cardiac thrombosis [54]. Importantly, cancer cell-produced plasminogen activator inhibitor-1 inhibits the fibrinolytic system, disrupting the pro-anticoagulation balance and facilitating thrombus formation [54]. Apart from the abovementioned intrinsic mechanisms, various factors, such as the site of malignancy (including hematological malignancies, lung, pancreas, or brain cancers), the presence of metastatic disease, and demographic patient characteristics may contribute to an increased incidence of thrombotic events in cancer patients [54,55].

### 3.7. Peripartum Cardiomyopathy

Peripartum cardiomyopathy (PPCM) is an infrequent cardiomyopathy variant manifesting in the late stages of pregnancy or postpartum, with notable features including compromised cardiac systolic function, diminished left ventricular ejection fraction (usually left ventricle ejection fractions < 45% but not limited to it), and/or the enlargement of the left ventricle, in the absence of identifiable alternative causes of heart failure [55]. Risk factors attributed to PPCM include African–American ethnicity, an older maternal age, multiple gestational pregnancy, and the presence of arterial hypertension [56]. Other potential contributors/mechanisms attributed to PPCM onset encompass various genetic factors, low selenium levels, viral infections during pregnancy, stress-triggered cytokines’ release and/or ongoing inflammation, an abnormal autoimmune response, pathological reactions to hemodynamic strain, disproportionate oxidative stress, and the stimulation of antiangiogenic factors, promoting endothelial injury [57].

The PPCM occurrence varies significantly based on the ethnic/racial and geographical context of women. Individuals of African descent, including Africans and African–Americans, face a heightened risk of PPCM, with estimated incidences of 1:100 pregnancies in Nigeria and 1:299 in Haiti; meanwhile, among Caucasian European populations, the incidences range from 1:1500 pregnancies in Germany to 1:10,000 in Denmark [57].

LVT was reported in approximately 10% to 17% of initial TTEs, with thromboembolic complications reported in 5% to 9% of affected women [56]. The high occurrence of thromboembolic events in PPCM was potentially associated with pregnancy-induced hypercoagulability, biventricular enlargement and dysfunction, venous stasis, prolonged bed rest, and postoperative conditions following cesarean section, with some of the abovementioned mechanisms being the substrate of PPCM development itself [56]. In addition to LVT onset, the presence of other risk factors, such as obesity, severe left ventricle systolic dysfunction (left ventricle ejection fraction ≤ 30%), and right ventricle abnormalities was linked to worse prognoses and incomplete recovery of cardiac function with high mortality rates [56,58], emphasizing the need for systematic screening of cardiac abnormalities, including the presence of intracavitary thrombi in patients at risk of PPCM occurrence.

In a recent study involving 159 suspected cases of PPCM in a Chinese cohort, intracardiac thrombi were identified in 22 patients, with 19 of them exhibiting LVT and 2 patients with biventricular thrombi [59]. The management in these cases is still debatable. Efficient and precise anticoagulation therapy holds considerable importance for PPCM patients, although the exact criteria for initiating such treatment remain uncertain [56,59]. In accordance with the Heart Failure Association of the European Society of Cardiology’s position statement on PPCM, a preventive regimen of low-molecular-weight heparin or oral anticoagulants is advised for PPCM patients with reduced left ventricular ejection fraction (LVEF) [57]. However, therapeutic anticoagulation is strongly recommended for those with confirmed intracardiac thrombus via imaging or evidence of systemic embolism, as well as for patients experiencing paroxysmal or persistent atrial fibrillation [57].

## 4. Pharmacological Treatment and Prevention

The primary goal of treatment is to prevent LVT occurrence. Most data on LVT and non-ischemic cardiomyopathy come from retrospective studies, and there is currently no recommendation on the use of prophylactic anticoagulation, although some authors suggest the possibility of starting therapeutic anticoagulation in certain conditions, especially in HCM with medium to large apical aneurysms [39].

Existing data on prophylactic anticoagulation in ischemic heart disease are not directly applicable to non-ischemic cardiomyopathy (NICM) patients, as these patients are also on antiplatelet therapy and have a different bleeding risk. The use of reduced-dose direct oral anticoagulants (DOACs) after a previous myocardial infarction to reduce the occurrence of left ventricular thrombus is being studied in the French APERITIF study (N = 560 patients; NCT05077683), in which a low dose of rivaroxaban at 2.5 mg twice daily is being evaluated [60]. This strategy seems promising, as shown in a study by Zhang Z et al., in which 279 patients with anterior ST-segment elevation myocardial infarction undergoing primary percutaneous coronary intervention were randomized to receive either low-dose rivaroxaban (2.5 mg twice daily) in addition to dual antiplatelet therapy (DAPT) or DAPT alone [61]. The primary endpoint was the occurrence of LVT within 30 days, and the results were positive, showing a reduction in thrombus formation (0.7% vs. 8.6%; HR: 0.08; 95% CI: 0.01–0.62; *p* = 0.015; *p* < 0.001 for superiority) [61].

After the formation of the thrombus, the objectives of antithrombotic treatment are to achieve the complete disappearance of the thrombus, prevent strokes and systemic embolism, and improve prognosis. In addition to a reduced risk of embolism, the association between complete resolution of the thrombus and a reduction in mortality was demonstrated in a recent study by Lattuca B et al., including 159 patients with diagnosed LVT [62]. Patients in this study were treated with vitamin K antagonists (VKAs) (48.4%), parenteral heparins (27.7%), and direct oral anticoagulants (22.6%). The frequency of events was significant, with a rate of major adverse cardiovascular events of 37.1%, mortality of 18.9%, stroke of 13.3%, and major bleeding of 13.2% during the follow-up period [62].

In the case of LVT’s onset in the course of an acute coronary syndrome, the latest guidelines from the European Society of Cardiology in 2023 on ACS management recommend starting oral anticoagulant therapy (warfarin or DOAC) once a left ventricular thrombus has been diagnosed for a duration of 3 to 6 months guided using repeated echocardiography or cardiac magnetic resonance imaging, taking into account the bleeding risk and the need for concomitant antiplatelet therapy [63].

The traditional treatment for intraventricular thrombus involves the use of VKAs to maintain an international normalized ratio (INR) between 2 and 3. The current scientific data on the use of VKAs are summarized mainly in a meta-analysis from the early 1990s based on only 270 patients in 7 studies. This analysis concluded that VKA treatment, compared to placebo, reduced the risk of embolic complications. However, there was no information regarding thrombus reduction [64]. Due to the difficulty of using VKAs and their challenging monitoring, the use of direct oral anticoagulants has been considered due to their favorable benefit–risk balance compared to VKAs in atrial fibrillation and venous thromboembolic disease. A recent study showed that up to 43.9% of patients diagnosed with LVT were treated with DOACs outside atrial fibrillation and venous thromboembolism indications [65].

The comparison of the effectiveness of DOAC with VKA in LVT treatment was studied in a meta-analysis by Saleh et al. [66]. Based on 13 studies and including 2395 patients, this meta-analysis highlighted similar regression rates between direct oral anticoagulants and VKAs, with rates of 71.4% and 71.9%, respectively (*p* = 0.36), and similarly high rates of embolic complications in line with the Lattuca B et al. cohort, with 15.6% and 21.3% of embolic events, respectively (*p* = 0.57) [66,67]. Furthermore, a prospective, multicenter, randomized study in 2021 by Alcalai et al. compared apixaban and warfarin in patients with LVT occurrence post-myocardial infarction [68]. Thirty-five participants were enrolled, demonstrating comparable thrombus regression rates at 3 months: 93% for warfarin and 94% for apixaban [68].

Lastly, in 2021, the No-LVT Trial led by Abdenabi et al. randomized 79 patients to receive either warfarin or rivaroxaban 20 mg for anticoagulation [67]. Of these patients, 78.5% had ischemic heart disease, and 53.1% were on dual antiplatelet therapy. Complete LVT resolution was observed in 71.79%, 76.92%, and 87.17% of the rivaroxaban group, compared to 47.5%, 67.5%, and 80% in the warfarin group at 1, 3, and 6 months, respectively [67]. Notably, resolution at one month was significantly higher in the rivaroxaban group compared to the warfarin group (odds ratio: 2.8; *p* = 0.03) [67]. DOACs appear to be a promising alternative to traditional oral anticoagulants (VKAs), especially in reducing bleeding risk. The doses of rivaroxaban and apixaban in these two studies are identical to those evaluated for atrial fibrillation doses used for preventing the formation of blood clots. Since patients with LVT are already in a thrombotic state, the use of a higher dosage derived from the treatment of venous thromboembolic disease is questionable. There is currently no study answering this question. Moreover, as the majority of patients are also treated with dual antiplatelet therapy, the use of higher doses of DOACs could pose a bleeding risk and result in a loss of benefit. The necessity of starting low-molecular-weight heparin for 7 days before switching to Dabigatran and Edoxaban is unknown. Additional studies are needed for this specific situation involving the presence of intracavitary thrombi.

### Future Clinical and Research Perspectives

Future investigations should prioritize establishing specific criteria for prophylactic or therapeutic anticoagulation to address intracavitary thrombi, tailored to the severity of cardiac dysfunction and additional risk factors impacting patient outcomes. The existing clinical protocols for anticoagulation therapy, even in the presence of atrial fibrillation, are subjected to numerous limitations, necessitating validation through large-scale, multi-ethnic, and multicentric cohorts.

While DOACs offer an alternative to VKAs, substantial gaps persist regarding their long-term efficacy in non-ischemic cardiomyopathies, including various pharmacological interactions that may alter their benefit. The uniqueness of our review lies in its comprehensive exploration of both ischemic and non-ischemic cardiopathies, encompassing cardiotoxicity—an area where current guidelines lack robust clinical evidence and often rely on expert consensus.

## 5. Conclusions

Left ventricular thrombus may occur in both ischemic and non-ischemic cardiomyopathies. While its incidence during acute coronary syndrome has decreased due to advancements like percutaneous angioplasty, it still ranges from 12 to 27% in anterior infarctions. The presence of LVT worsens prognoses, increasing the risks of death, stroke, systemic embolism, and bleeding related to treatment. Although late gadolinium-enhanced cardiac magnetic resonance imaging is the favored diagnostic approach, its limited accessibility poses challenges in clinical practice. There are epidemiological distinctions between LVT associated with ischemic cardiomyopathy and non-ischemic cardiomyopathy. However, determining LVT’s prevalence in various cardiopathies (such as Takotsubo syndrome, hypertrophic cardiomyopathy with apical aneurysm, eosinophilic myocarditis, Chagas disease, and amyloidosis) remains challenging due to limitations in study design, their retrospective nature, and biases inherent in registry or small retrospective studies. The current treatment still relies on the use of VKAs, although several studies and meta-analyses tendto generalize the use of DOACs. There are currently no prospective randomized data on the optimal treatment regimen, duration of anticoagulation, or the combination of oral anticoagulation with antiplatelet agents, so the choice of treatment should be adapted to the patient’s clinical condition and follow-up.

## References

[1] Habash F., Vallurupalli S. (2017). Challenges in Management of Left Ventricular Thrombus. Ther. Adv. Cardiovasc. Dis..

[2] Lee J.M., Park J.J., Jung H.W., Cho Y.S., Oh I.Y., Yoon C.H., Suh J.-W., Chun E.J., Choi S.I., Youn T.-J. (2013). Left Ventricular Thrombus and Subsequent Thromboembolism, Comparison of Anticoagulation, Surgical Removal, and Antiplatelet Agents. J. Atheroscler. Thromb..

[3] Johannessen K.A., Nordrehaug J.E., Von Der Lippe G. (1984). Left Ventricular Thrombosis and Cerebrovascular Accident in Acute Myocardial Infarction. Br. Heart J..

[4] Lamas G.A., Vaughan D.E., Pfeffer M.A. (1988). Left Ventricular Thrombus Formation after First Anterior Wall Acute Myocardial Infarction. Am. J. Cardiol..

[5] Asinger R.W., Mikell F.L., Elsperger J., Hodges M. (1981). Incidence of Left-Ventricular Thrombosis after Acute Transmural Myocardial Infarction. Serial Evaluation by Two-Dimensional Echocardiography. N. Engl. J. Med..

[6] Shacham Y., Leshem-Rubinow E., Ben Assa E., Rogowski O., Topilsky Y., Roth A., Steinvil A. (2013). Frequency and Correlates of Early Left Ventricular Thrombus Formation Following Anterior wall Acute Myocardial Infarction Treated with Primary Percutaneous Coronary Intervention. Am. J. Cardiol..

[7] Osherov A.B., Borovik-Raz M., Aronson D., Agmon Y., Kapeliovich M., Kerner A., Grenadier E., Hammerman H., Nikolsky E., Roguin A. (2009). Incidence of Early Left Ventricular Thrombus after Acute Anterior Wall Myocardial Infarction in the Primary Coronary Intervention Era. Am. Heart J..

[8] Solheim S., Seljeflot I., Lunde K., Bjørnerheim R., Aakhus S., Forfang K., Arnesen H. (2010). Frequency of Left Ventricular Thrombus in Patients with Anterior Wall Acute Myocardial Infarction Treated with Percutaneous Coronary Intervention and Dual Antiplatelet Therapy. Am. J. Cardiol..

[9] Cambronero-Cortinas E., Bonanad C., Monmeneu J.V., Lopez-Lereu M.P., Gavara J., De Dios E., Rios C., Perez N., Racugno P., Paya A. (2017). Incidence, Outcomes, and Predictors of Ventricular Thrombus after Reperfused ST-Segment-Elevation Myocardial Infarction by Using Sequential Cardiac MR Imaging. Radiology.

[10] Weir R.A.P., Martin T.N., Petrie C.J., Murphy A., Clements S., Steedman T., Wagner G.S., McMurray J.J., Dargie H.J. (2009). Cardiac and Extracardiac Abnormalities Detected by Cardiac Magnetic Resonance in a Post-Myocardial Infarction Cohort. Cardiology.

[11] Bière L., Audonnet M., Clerfond G., Delagarde H., Willoteaux S., Prunier F., Furber A. (2016). First Pass Perfusion Imaging to Improve the Assessment of Left Ventricular Thrombus Following a Myocardial Infarction. Eur. J. Radiol..

[12] Massussi M., Scotti A., Lip G.Y.H., Proietti R. (2021). Left Ventricular Thrombosis: New Perspectives on an Old Problem. Eur. Heart J. Cardiovasc. Pharmacother..

[13] Ding K.J., Cammann V.L., Szawan K.A., Stähli B.E., Wischnewsky M., Di Vece D., Citro R., Jaguszewski M., Seifert B., Sarcon A. (2020). Intraventricular Thrombus Formation and Embolism in Takotsubo Syndrome: Insights From the International Takotsubo Registry. Arterioscler. Thromb. Vasc. Biol..

[14] Santoro F., Stiermaier T., Tarantino N., De Gennaro L., Moeller C., Guastafierro F., Marchetti M.F., Montisci R., Carapelle E., Graf T. (2017). Left Ventricular Thrombi in Takotsubo Syndrome: Incidence, Predictors, and Management: Results from the GEIST (German Italian Stress Cardiomyopathy) Registry. J. Am. Heart Assoc..

[15] Hooks M., Okasha O., Velangi P.S., Nijjar P.S., Farzaneh-Far A., Shenoy C. (2020). Left Ventricular Thrombus on Cardiovascular Magnetic Resonance Imaging in Non-Ischaemic Cardiomyopathy. Eur. Heart J. Cardiovasc. Imaging.

[16] Jug B., Vene N., Salobir B.G., Šebeštjen M., Šabovič M., Keber I. (2009). Procoagulant State in Heart Failure with Preserved Left Ventricular Ejection Fraction. Int. Heart J..

[17] Acar Z., Ziyrek M., Korkmaz L., Kiris A., Sahin S., Celik S. (2014). Mean Platelet Volume at Admission Is a Determinant of Left Ventricular Thrombus Formation after Primary Percutaneous Coronary Intervention for First Anterior Wall Myocardial Infarction. Acta Cardiol..

[18] Camaj A., Fuster V., Giustino G., Bienstock S.W., Sternheim D., Mehran R., Dangas G.D., Kini A., Sharma S.K., Halperin J. (2022). Left Ventricular Thrombus Following Acute Myocardial Infarction: JACC State-of-the-Art Review. J. Am. Coll. Cardiol..

[19] Funke Küpper A.J., Verheugt F.W.A., Peels C.H., Galema T.W., Roos J.P. (1989). Left Ventricular Thrombus Incidence and Behavior Studied by Serial Two-Dimensional Echocardiography in Acute Anterior Myocardial Infarction: Left Ventricular Wall Motion, Systemic Embolism and Oral Anticoagulation. J. Am. Coll. Cardiol..

[20] Nihoyannopoulos P., Smith G.C., Maseri A., Foale R.A. (1989). The Natural History of Left Ventricular Thrombus in Myocardial Infarction: A Rationale in Support of Masterly Inactivity. J. Am. Coll. Cardiol..

[21] Motro M., Barbash G.I., Hod H., Roth A., Kaplinsky E., Laniado S., Keren G. (1991). Incidence of Left Ventricular Thrombi Formation after Thrombolytic Therapy with Recombinant Tissue Plasminogen Activator, Heparin, and Aspirin in Patients with Acute Myocardial Infarction. Am. Heart J..

[22] Ram P., Shah M., Sirinvaravong N., Lo K.B., Patil S., Patel B., Tripathi B., Garg L., Figueredo V. (2018). Left Ventricular Thrombosis in Acute Anterior Myocardial Infarction: Evaluation of Hospital Mortality, Thromboembolism, and Bleeding. Clin. Cardiol..

[23] Meurin P., Brandao Carreira V., Dumaine R., Shqueir A., Milleron O., Safar B., Perna S., Smadja C., Genest M., Garot J. (2015). Incidence, diagnostic methods, and evolution of left ventricular thrombus in patients with anterior myocardial infarction and low left ventricular ejection fraction: A prospective multicenter study. Am. Heart J..

[24] Garg P., Van Der Geest R.J., Swoboda P.P., Crandon S., Fent G.J., Foley J.R.J., Dobson L.E., Al Musa T., Onciul S., Vijayan S. (2019). Left ventricular thrombus formation in myocardial infarction is associated with altered left ventricular blood flow energetics. Eur. Heart J. Cardiovasc. Imaging.

[25] Stein B., Fuster V. (1989). Antithrombotic therapy in acute myocardial infarction: Prevention of venous, left ventricular and coronary artery thromboembolism. Am. J. Cardiol..

[26] Saric M., Armour A.C., Arnaout M.S., Chaudhry F.A., Grimm R.A., Kronzon I., Landeck B.F., Maganti K., Michelena H.I., Tolstrup K. (2016). Guidelines for the Use of Echocardiography in the Evaluation of a Cardiac Source of Embolism. J. Am. Soc. Echocardiogr..

[27] Roifman I., Connelly K.A., Wright G.A., Wijeysundera H.C. (2015). Echocardiography vs. Cardiac Magnetic Resonance Imaging for the Diagnosis of Left Ventricular Thrombus: A Systematic Review. Can. J. Cardiol..

[28] Bulluck H., Chan M.H.H., Paradies V., Yellon R.L., Ho H.H., Chan M.Y., Chin C.W.L., Tan J.W., Hausenloy D.J. (2018). Incidence and Predictors of Left Ventricular Thrombus by Cardiovascular Magnetic Resonance in Acute ST-Segment Elevation Myocardial Infarction Treated by Primary Percutaneous Coronary Intervention: A Meta-Analysis. J. Cardiovasc. Magn. Reson..

[29] Weinsaft J.W., Kim J., Medicherla C.B., Ma C.L., Codella N.C.F., Kukar N., Alaref S., Kim R.J., Devereux R.B. (2016). Echocardiographic Algorithm for Post-Myocardial Infarction LV Thrombus: A Gatekeeper for Thrombus Evaluation by Delayed Enhancement CMR. JACC Cardiovasc. Imaging.

[30] Gellen B., Biere L., Logeart D., Lairez O., Vicaut E., Furber A., Mercadier J.-J., Sirol M. (2017). Timing of Cardiac Magnetic Resonance Imaging Impacts on the Detection Rate of Left Ventricular Thrombus After Myocardial Infarction. JACC Cardiovasc Imaging.

[31] Arbelo E., Protonotarios A., Gimeno J.R., Arbustini E., Barriales-Villa R., Basso C., Bezzina C.R., Biagini E., Blom N.A., de Boer R.A. (2023). 2023 ESC Guidelines for the management of cardiomyopathies: Developed by the task force on the management of cardiomyopathies of the European Society of Cardiology (ESC). Eur. Heart J..

[32] Chimenti C., Lavalle C., Magnocavallo M., Alfarano M., Mariani M.V., Bernardini F., Della Rocca D.G., Galardo G., Severino P., Di Lullo L. (2022). A Proposed Strategy for Anticoagulation Therapy in Noncompaction Cardiomyopathy. ESC Heart Fail..

[33] Lehmonen L., Putaala J., Pöyhönen P., Kuusisto J., Pirinen J., Sinisalo J., Järvinen V. (2022). MRI-Derived Cardiac Washout Is Slowed in the Left Ventricle and Associated with Left Ventricular Non-Compaction in Young Patients with Cryptogenic Ischemic Stroke. Int. J. Cardiovasc. Imaging.

[34] Costantino J., Ajmone F.M., Maggio E., Ballatore F., Manguso G., Ciaramella P., Galea N., Alfarano M., Severino P., Lavalle C. (2023). Anticoagulant Therapy in Left Ventricular Non-Compaction: When, How and Why. G. Ital. Cardiol..

[35] Baldetti L., Pagnesi M., Gallone G., Beneduce A., Belardinelli P., Melillo F., Spoladore R., Latib A., Colombo A., Giannini F. (2019). Thrombotic Complications and Cerebrovascular Events in Takotsubo Syndrome: A Systematic Review and Meta-Analysis. Can. J. Cardiol..

[36] Rowin E.J., Maron B.J., Haas T.S., Garberich R.F., Wang W., Link M.S., Maron M.S. (2017). Hypertrophic Cardiomyopathy with Left Ventricular Apical Aneurysm: Implications for Risk Stratification and Management. J. Am. Coll. Cardiol..

[37] Papanastasiou C.A., Zegkos T., Karamitsos T.D., Rowin E.J., Maron M.S., Parcharidou D., Kokkinidis D.G., Karvounis H., Rimoldi O., Maron B.J. (2021). Prognostic Role of Left Ventricular Apical Aneurysm in Hypertrophic Cardiomyopathy: A Systematic Review and Meta-Analysis. Int. J. Cardiol..

[38] Lee D.Z.J., Montazeri M., Bataiosu R., Hoss S., Adler A., Nguyen E.T., Rakowski H., Chan R.H. (2022). Clinical Characteristics and Prognostic Importance of Left Ventricular Apical Aneurysms in Hypertrophic Cardiomyopathy. JACC Cardiovasc. Imaging.

[39] Caforio A.L.P., Pankuweit S., Arbustini E., Basso C., Gimeno-Blanes J., Felix S.B., Fu M., Heliö T., Heymans S., Jahns R. (2013). Current State of Knowledge on Aetiology, Diagnosis, Management, and Therapy of Myocarditis: A Position Statement of the European Society of Cardiology Working Group on Myocardial and Pericardial Diseases. Eur. Heart J..

[40] Lampejo T., Durkin S.M., Bhatt N., Guttmann O. (2021). Acute Myocarditis: Aetiology, Diagnosis and Management. Clin. Med..

[41] Brambatti M., Matassini M.V., Adler E.D., Klingel K., Camici P.G., Ammirati E. (2017). Eosinophilic Myocarditis: Characteristics, Treatment, and Outcomes. J. Am. Coll. Cardiol..

[42] Umezawa E.S., Stolf A.M.S., Corbett C.E.P., Shikanai-Yasuda M.A. (2001). Chagas’ Disease. Lancet.

[43] Nunes M.C.P., Beaton A., Acquatella H., Bern C., Bolger A.F., Echeverría L.E., Dutra W.O., Gascon J., Morillo C.A., Oliveira-Filho J. (2018). Chagas Cardiomyopathy: An Update of Current Clinical Knowledge and Management: A Scientific Statement from the American Heart Association. Circulation.

[44] WHO Expert Committee (2002). Control of Chagas Disease. World Health Organ. Tech. Rep. Ser..

[45] Carod-Artal F.J., Vargas A.P., Horan T.A., Nunes L.G.N. (2005). Chagasic Cardiomyopathy Is Independently Associated with Ischemic Stroke in Chagas Disease. Stroke.

[46] Moreira H.T., Volpe G.J., Mesquita G.M., Braggion-Santos M.F., Pazin-Filho A., Marin-Neto J.A., Schmidt A. (2022). Association of Left Ventricular Abnormalities with Incident Cerebrovascular Events and Sources of Thromboembolism in Patients with Chronic Chagas Cardiomyopathy. J. Cardiovasc. Magn. Reson..

[47] Feng D.L., Syed I.S., Martinez M., Oh J.K., Jaffe A.S., Grogan M., Edwards W.D., Gertz M.A., Klarich K.W. (2009). Intracardiac Thrombosis and Anticoagulation Therapy in Cardiac Amyloidosis. Circulation.

[48] Martinez-Naharro A., Gonzalez-Lopez E., Corovic A., Mirelis J.G., Baksi A.J., Moon J.C., Garcia-Pavia P., Gillmore J.D., Hawkins P.N., Fontana M. (2019). High Prevalence of Intracardiac Thrombi in Cardiac Amyloidosis. J. Am. Coll. Cardiol..

[49] Lyon A.R., López-Fernánde T., Couch L.S., Asteggiano R., Aznar M.C., Bergler-Klei J., Boriani G., Cardinale D., Cordoba R., Cosyns B. (2022). 2022 ESC Guidelines on Cardio-Oncology Developed in Collaboration with the European Hematology Association (EHA), the European Society for Therapeutic Radiology and Oncology (ESTRO) and the International Cardio-Oncology Society (IC-OS): Developed by the task force on cardio-oncology of the European Society of Cardiology (ESC). Eur. Heart J..

[50] Grover S.P., Hisada Y.M., Kasthuri R.S., Reeves B.N., MacKman N. (2021). Cancer Therapy–Associated Thrombosis. Arterioscler. Thromb. Vasc. Biol..

[51] Roy R., Guile B., Sun D., Szasz T., Singulane C.C., Nguyen D., Abutaleb A., Lang L.M., Addetia K. (2024). Right Ventricular Thrombus on Echocardiography. Am. J. Cardiol..

[52] Ikeda A., Yamachika E., Mizutani M., Matsubara M., Moritani N., Nakatsuji K., Iida S. (2017). Rapid Occurrence of Left Ventricular Thrombus Associated with Platinum-Based Chemotherapy Plus Cetuximab for the Treatment of Metastatic Squamous Cell Carcinoma of the Head and Neck: A Case Report. Mol. Clin. Oncol..

[53] Kitkungvan D., Yusuf S.W., Moudgil R., Palaskas N., Guindani M., Juhee S., Hassan S., Sanchez L., Banchs J. (2018). Echocardiographic Measures Associated with the Presence of Left Ventricular Thrombus in Patients with Chemotherapy-Related Cardiac Dysfunction. Echocardiography.

[54] Fernandes C.J., Morinaga L.T.K., Alves J.L., Castro M.A., Calderaro D., Jardim C.V.P., Souza R. (2019). Cancer-Associated Thrombosis: The When, How and Why. Eur. Respir. Rev..

[55] Alizadehasl A., Saedi T., Saedi S. (2021). Right Ventricular Clot in Renal Cell Carcinoma A Huge Right Ventricular Thrombus Clot in Renal Cell Carcinoma Without Inferior Vena Cava Involvement. Iran. Heart J..

[56] Davis M.B., Arany Z., McNamara D.M., Goland S., Elkayam U. (2020). Peripartum Cardiomyopathy: JACC State-of-the-Art Review. J. Am. Coll. Cardiol..

[57] Bauersachs J., König T., van der Meer P., Petrie M.C., Hilfiker-Kleiner D., Mbakwem A., Hamdan R., Jackson A.M., Forsyth P., de Boer R.A. (2019). Pathophysiology, Diagnosis and Management of Peripartum Cardiomyopathy: A Position Statement from the Heart Failure Association of the European Society of Cardiology Study Group on Peripartum Cardiomyopathy. Eur. J. Heart Fail..

[58] Amos A.M., Jaber W.A., Russell S.D. (2006). Improved outcomes in peripartum cardiomyopathy with contemporary. Am. Heart J..

[59] Fu K., Zhang H., Chen N., Hu Y., Xiao J., Zhang X., Lin Z., Lu H., Ji X. (2023). Risk factors for intracardiac thrombus in peripartum cardiomyopathy: A retrospective study in China. ESC Heart Fail..

[60] Puymirat E., Soulat G., Fayol A., Mousseaux E., Montalescot G., Cayla G., Steg P.G., Berard L., Rousseau A., Drouet É. (2023). Rationale and Design of the Direct Oral Anticoagulants for Prevention of Left Ventricular Thrombus after Anterior Acute Myocardial Infarction (APERITIF) Trial. Am. Heart J..

[61] Zhang Z., Si D., Zhang Q., Jin L., Zheng H., Qu M., Yu M., Jiang Z., Li D., Li S. (2022). Prophylactic Rivaroxaban Therapy for Left Ventricular Thrombus after Anterior ST-Segment Elevation Myocardial Infarction. JACC Cardiovasc. Interv..

[62] Lattuca B., Bouziri N., Kerneis M., Portal J.J., Zhou J., Hauguel-Moreau M., Mameri A., Zeitouni M., Guedeney P., Hammoudi N. (2020). Antithrombotic Therapy for Patients With Left Ventricular Mural Thrombus. J. Am. Coll. Cardiol..

[63] Byrne R.A., Rossello X., Coughlan J.J., Barbato E., Berry C., Chieffo A., Mameri A., Zeitouni M., Guedeney P., Hammoudi N. (2023). 2023 ESC Guidelines for the Management of Acute Coronary Syndromes. Eur. Heart J..

[64] Vaitkus P.T., Barnathan E.S. (1993). Embolic Potential, Prevention and Management of Mural Thrombus Complicating Anterior Myocardial Infarction: A Meta-Analysis. J. Am. Coll. Cardiol..

[65] Robinson A.A., Trankle C.R., Eubanks G., Schumann C., Thompson P., Wallace R.L., Gottiparthi S., Ruth B., Kramer C.M., Salerno M. (2020). Off-label Use of Direct Oral Anticoagulants Compared With Warfarin for Left Ventricular Thrombi. JAMA Cardiol..

[66] Saleh Y., Al-abcha A., Abdelkarim O., Abdelnabi M., Almaghraby A. (2021). Meta-Analysis Investigating the Role of Direct Oral Anticoagulants Versus Vitamin K Antagonists in the Treatment of Left Ventricular Thrombi. Am. J. Cardiol..

[67] Abdelnabi M., Saleh Y., Fareed A., Nossikof A., Wang L., Morsi M., Eshak N., Abdelkarim O., Badran H., Almaghraby A. (2021). Comparative Study of Oral Anticoagulation in Left Ventricular Thrombi (No-LVT Trial). J. Am. Coll. Cardiol..

[68] Alcalai R., Butnaru A., Moravsky G., Yagel O., Rashad R., Ibrahimli M., Planer D., Amir O., Elbaz-Greener G., Leibowitz D. (2022). Apixaban vs. Warfarin in Patients with Left Ventricular Thrombus: A Prospective Multicentre Randomized Clinical Trial‡. Eur. Heart J. Cardiovasc. Pharmacother..

